# Longitudinal evolution of the immune suppressive glioma microenvironment in different synchronous lesions during treatment

**DOI:** 10.1093/noajnl/vdz053

**Published:** 2020-01-20

**Authors:** Susanna Mandruzzato, Laura Pinton, Elena Masetto, Marina Vettore, Camilla Bonaudo, Giuseppe Lombardi, Alessandro della Puppa

**Affiliations:** 1 Department of Surgery, Oncology and Gastroenterology, University of Padova, Padova; 2 Immunology and Molecular Oncology Unit, Veneto Institute of Oncology IOV-IRCCS, Padova; 3 Department of Oncology, Oncology 1, Veneto Institute of Oncology IOV-IRCCS, Padova; 4 Neurosurgery Department, University Hospital, Padova

The role of immune suppression in glioma progression has been clearly established.^1^ We and others have recently demonstrated that myeloid cells play a major role in the tumor microenvironment of glioblastoma (GBM) patients,^2,3^ and that not only bone marrow-derived macrophages (BMDMs) have a higher intrinsic immune suppressive ability compared to resident microglial cells (MG), but also that this ability greatly increases going from the periphery to the tumor core.^3^ In lower grade gliomas (grades II and III), a much lower amount of BMDM is present, devoid of immune suppressive ability.^3^ We present here a longitudinal analysis of the immune infiltrate in a patient with a synchronous occurrence of GBM in the left temporal lobe, and a low-grade glioma (LGG) in the right frontal lobe, with discordant isocitrate dehydrogenase (IDH)-mutational status,^4^ followed by two GBM relapses.

## Case Report Presentation Results

### Clinical History and Histological Findings

**Figure 1.**
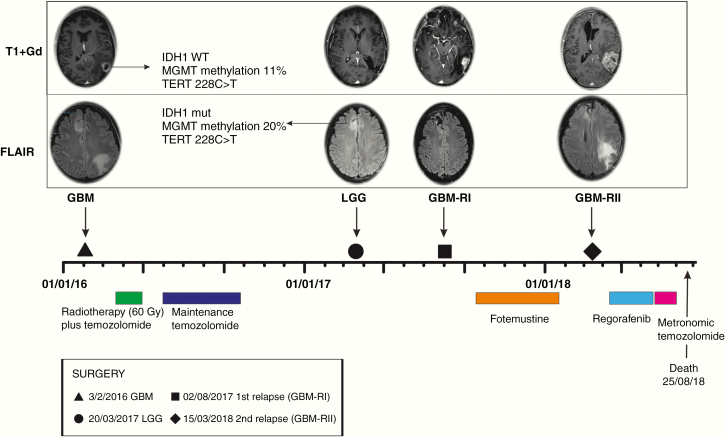
Timeline of surgical and pharmacological treatments. The upper panel shows the radiological and molecular characteristics of GBM, LGG, first relapse (GBM-RI), and second relapse (GBM-RII).

A 48-year-old female patient, with no previous history of cancer, was admitted to the Neurosurgical Department at the Hospital of Padua with clinical signs and symptoms, such as aphasia, headache, disorientation in time and space, and scotomas. Computed tomography (CT) and Magnetic Resonance Imaging (MRI) scans showed the enhancing left temporal lesion and the right frontal lesion in the right superior frontal gyrus (Figure 1). In February 2016 a surgical excision of the lesion in the left temporal lobe was performed, using the intraoperative navigation system, with 5-Aminolevulinic-acid-induced-fluorescence (5-ALA). The histological and molecular examination of the left temporal lesion demonstrated an IDH wild-type (wt) GBM (grade IV, WHO 2016). The neuroimaging controls showed a macroscopically complete excision of the tumor and the persistence of the frontal lesion. Subsequently, concomitant temozolomide and radiotherapy followed by 6 cycles of maintenance temozolomide, according to Stupp protocol, were administered.^5^

After 1 year from the first operation, due to stable disease, a second surgery was performed in order to remove the right non-enhancing frontal lesion. The histological diagnosis was IDH1-mutant diffuse astrocytoma (grade II, WHO 2016), macroscopically removed as the following CT and MRI scans documented. No chemotherapy or radiotherapy was administered after the second surgery.

In July 2017, a new MRI scan documented a relapse of the lesion in the temporal lobe, and in August 2017 the surgical excision of the recurrent tumor was performed. A second-line chemotherapy with fotemustine was administered until January 2018 when a new relapse was documented by a brain MRI. On March 2018, a new surgical removal was performed, using 5-ALA fluorescence. The histological examination confirmed the diagnosis of recurrent GBM IDH wt. After surgery, a third-line treatment with regorafenib and subsequently, fourth-line therapy with metronomic temozolomide (TMZ) were administered. The residual component of the lesion was increased dimensionally on the last MRI scan, performed in June 2018, and the patient died in August 2018.

### Molecular Characterization of the Multifocal Glioma

Both GBM and LGG lesions were characterized molecularly, as previously described.^4^ Briefly, the lesion in the left temporal lobe was diagnosed as GBM IDH wt (grade IV WHO 2016) at the histological exam, whereas the concomitant lesion in the superior frontal gyrus was diagnosed as diffuse astrocytoma carrying IDH1 mutation.^4^ Only the GBM lesion, analyzed by fluorescence-in situ-hybridization, carried monosomy of chromosome 10 and homozygous deletion of 9p21 (cyclin-dependent kinase inhibitor 2A gene [CDKN2A]).^4^ Also, the expression of DNA mismatch repair (MMR) proteins was investigated, showing that both lesions were MMR proficient. Furthermore, microsatellite instability was detected in both lesions, that also carried a Telomerase Reverse Transcriptase (TERT) promoter mutation (228C>T).

### Immunological Characterization of Blood and Tumor Lesions

**Figure 2.**
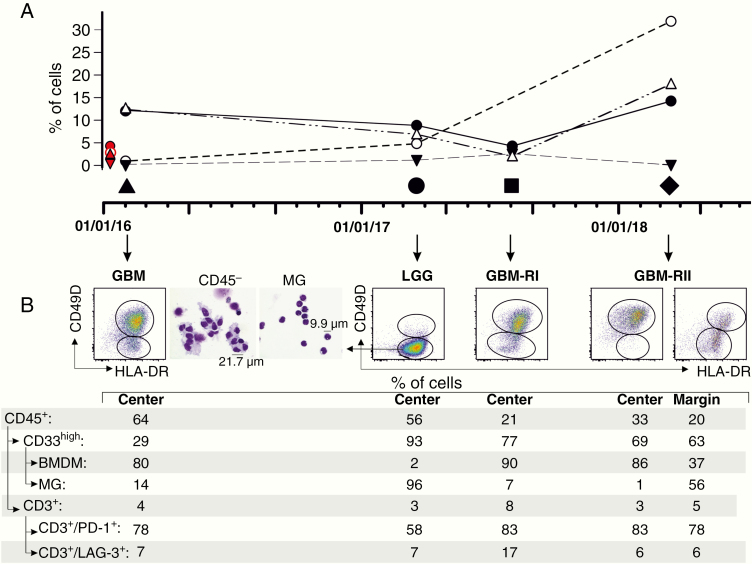
Immunophenotypical analysis. (A) Levels of blood MDSC-1 (CD14^+^/IL4Rα ^+^, ●), MDSC-2 (CD15^+^/IL4Rα ^+^, ○), MDSC-3 (Lin^−^/HLA-DR^−^/CD33^+^/CD11b^+^, ▽) and MDSC-4 (CD14^+^/HLA-DR^low/−^ △) performed immediately prior to surgery. Red symbols correspond to median levels of the same MDSC subset in a group of age-matched healthy donors. (B) Flow cytometry panels of CD49D^+^/HLA-DR^+^ cells gated on live CD45^+^/CD33^+^ cells (BMDM) and CD49D^−^/HLA-DR^+^ cells, gated on live CD45^+^/CD33^+^ cells (MG) in tumor layers identified by 5-ALA fluorescence. Cytospins were obtained by centrifuging 1 × 10^4^ sorted cells on microscope slides and stained with May-Grünwald and Giemsa dye. Cell morphology was examined by microscopic evaluation at 40× magnification and without immersion oil. In LGG, resection was performed with MR-assisted navigation.

Blood and the four clinical specimens were analyzed at each surgery as shown in Figure 2, to define the main parameters that characterize the immune suppression induced by myeloid cells. Tumor resection was guided with 5-ALA-induced-fluorescence and magnetic resonance (MR)-assisted navigation. A specimen was analyzed immediately after each resection from the brightly fluorescent area, to evaluate the leukocyte infiltrate by multicolor flow cytometry, and, in the fourth surgery two specimens were evaluated, from both the central and the peritumoral area (Figure 2). Peripheral blood was collected immediately prior to each surgery, and four myeloid-derived suppressor cell (MDSC) subsets were evaluated, monocytic MDSC-1 (CD14^+^/IL4Rα ^+^) and MDSC-4 (CD14^+^/HLA-DR^low/−^), polymorphonuclear (PMN)-MDSC-2 (CD15^+^/IL4Rα ^+^), and early stage MDSC-3 (Lin^−^/HLA-DR^−^/CD33^+^/CD11b^+^).^6^

In line with our previous results, we observed in the central area of GBM tissues from the first surgery and from both relapses a large proportion of BMDMs (ranging from 80% to 90% of total macrophages), and a lower amount of MG cells (from 1% to 14% of the total macrophages, Figure 2, panel B). In the second relapse, comparison of the marginal with the central area showed a reduced amount of BMDM and increased presence of MG cells (37% vs 56%, respectively). Conversely, in the low-grade astrocytoma despite the presence of a high percentage of CD33^+^ myeloid infiltrate, in which 96% of cells were MG (Figure 2, cytospin in panel B), there was not a detectable presence of immune suppressive BMDM. The presence of this type and percentage of myeloid infiltrate is in line with our previous results obtained from untreated LGG patients, while in this case the patient underwent a previous treatment (Figure 1), that did not alter significantly the recruitment of myeloid cells.

Of note, GBM lesion had an unusual presence of PMN, which accounted for 65% of cells. This population was present at lower levels in the other glioma samples analyzed from the same patient, and thus the higher levels of PMNs in GBM tissue explain the lower percentage of CD33^+^ macrophages in this sample.

Concerning the circulating myeloid cells, we observed over time an increased presence of MDSCs, and more precisely of monocytic subsets 1 and 4, and of PMN-MDSC-2,^6^ and a stable presence of early-stage MDSC-3 (Figure 2A,), thus supporting the view of an increased and altered myelopoiesis in the patient, driving the accumulation of suppressive BMDMs.

Following therapy, the percentage of tumor-infiltrating leukocytes decreased in the two relapses (GBM-RI and RII), but both lesions did not change the composition of the immune infiltrate, which was constituted mainly by CD33^+^ myeloid cells, and BMDMs accumulated in the center of the lesion of GBM-RII (Figure 2, panel B). The presence of CD3^+^ T cells did not change significantly over time and after therapies, or between high and low-grade lesions, showing the characteristics of a “cold” infiltrate, with a stable expression of dysfunctional markers PD-1 and LAG-3.

**Figure 3.**
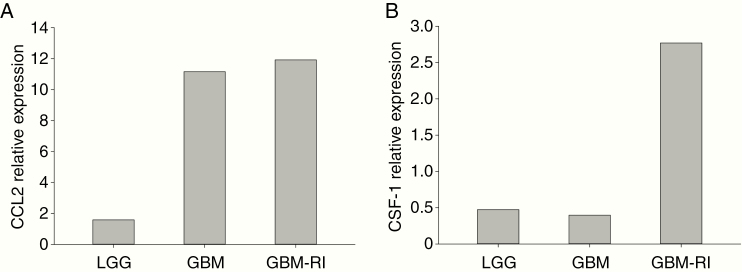
CCL2 and CSF-1 gene expression in glioma cells. CCL2 (panel A) and CSF-1 (panel B) gene expression levels were evaluated by real-time RT-PCR on CD45^−^ cells separated by FACS sorting from the cell suspension obtained from the tumor tissue corresponding to LGG, GBM, and GBM-RI.

Among the factors driving myeloid cells infiltration, chemoattractant CCL2 and CSF1/M-CSF1 play a relevant role.^7,8^ To evaluate their role in sustaining BMDM infiltration in low- and high-grade lesions, we sorted CD45^−^ cells from GBM, GBM-RI, and LGG lesions and assessed by quantitative real-time reverse transcription-polymerase chain reaction (qRT-PCR) the expression levels of these cytokines. As shown in Figure 3, GBM and GBM-RI lesions have a large increase of CCL2 expression compared to LGG lesion. This result is in line with the hypothesis that CCL2 released from tumor cells of high-grade glioma, but not LGG is responsible for the recruitment of BMDMs. CSF-1 showed no difference between LGG and GBM, but was expressed at higher level in GBM relapse. Since BMDMs are present both in GBM and GBM relapse, the pattern of expression of CSF-1 does not support a possible involvement of this cytokine in macrophage infiltration into tumor lesion.

## Discussion

In our previous work, we observed that tumor grading in gliomas influences the recruitment of BMDM, since LGG has a significant lower amount of these suppressive macrophages.^3^ So far, the simultaneous presence of low-grade and high-grade gliomas has never been explored from this point of view. Results from this unique case recapitulate the findings observed on a large number of cases, and indicate that when a simultaneous high- and low-grade lesions are present, each tumor microenvironment dictates the recruitment of the inflammatory cells. We found that CCL2, but not CSF-1 produced by tumor cells is highly expressed by high-grade lesions, thus highlighting the role of this chemokine in the recruitment of suppressive BMDMs. However, the integrity of the blood–brain barrier (BBB) may also play a role. In fact, the BBB is disrupted during GBM progression, and shows heterogeneous permeability, as evidenced by contrast-enhanced MRI, the gold standard method for assessing BBB dysfunction.^9,10^ The case that we present in this manuscript is in line with these observations, as GBM lesions show contrast enhancement, while the LGG lesion does not, after contrast medium injection.

The compromised integrity of the BBB may facilitate the passage of the monocytes, and the consequent accumulation and differentiation into suppressive BMDMs. Thus, the presence of a large myeloid infiltrate in high-grade gliomas suggests an active passage of immune cells, driven by chemoattractant CCL2 and by a leaky barrier. In the simultaneous low-grade lesion, instead, the lack of contrast enhancement suggests the presence of an intact BBB, which, coupled to low or absent presence of CCL2, may explain the absence of suppressive BMDM in the tumor microenvironment.

Our results indicate that the high-grade lesions (GBM, GBM-RI, and GBM-RII) show the typical presence and proportion of macrophages with a prevalence of recruited BMDM. The same holds true for the low-grade lesion, in which macrophages are represented almost exclusively by MG cells, as observed in patients in which a single lesion is present, indicating that the local recruitment of myeloid cells in each lesion is not influenced by the presence of the other lesion.

Results learned from this unique case stress the notion that blood-derived immune suppressive macrophages are a hallmark of high-grade gliomas, that their persistent accumulation in recurrent lesions, despite therapies, is an obstacle that must be overcome to find new efficient treatments, and finally that a rational therapeutic approach to GBM should include the targeting not only of BMDMs, but also of circulating myeloid cells, and of the soluble factors driving their accumulation.

## Funding

This work was supported by Italian Association for Cancer Research (AIRC) (IG2015-17400 to S.M.), TRANSCAN-2, ERA-NET to S.M. and Università degli Studi di Padova (CPDA144873/14 to S.M.).

## 

**Conflict of interest statement**. None declared.

## Authorship statement. 

S.M. and A.D.P designed the study and supervised the work. S.M. wrote the manuscript and handled funding. L.P., E.M., M.V. performed experiments and analyzed the data. G.L. provided clinical information and discussed the data. A.D.P. performed neurosurgery. All authors have read and approved the final version of the manuscript.
